# Dynamic response and failure mechanism of rural masonry structures impacted by debris flow containing boulders

**DOI:** 10.1371/journal.pone.0330247

**Published:** 2026-03-27

**Authors:** Yanan Fu, Enqiao Shi, Yujiang Wang

**Affiliations:** 1 Department of Civil Engineering, Chongqing Three Gorges University, Wanzhou, China; 2 Chongqing Engineering Research Center of Disaster Prevention & Control for Banks and Structures in Three Gorges Reservoir Area, Wanzhou, China; University of Zanjan, IRAN, ISLAMIC REPUBLIC OF

## Abstract

Rural masonry structures in mountainous regions are highly vulnerable to debris flow impacts. However, their dynamic failure mechanisms remain poorly quantified. To address this knowledge gap and enhance the debris flow resilience of such structures in the Three Gorges Reservoir Area, a Fluid-Structure Interaction model was established and validated using ANSYS Workbench. The model simulated the impact of debris flows containing boulders and incorporated the Drucker-Prager constitutive model for the masonry material. Parametric analysis revealed a bimodal dynamic response, characterized by the peak boulder force lagging behind the peak slurry force. A critical flow velocity of 5 m/s was identified: above this threshold, slurry force dominated (*F*_slurry_ > *F*_boulder_), while below it, the boulder force was greater (*F*_slurry_ < *F*_boulder_). The critical failure parameters were quantified as follows: At a flow depth of 1.5 m, the critical velocities for local and global collapse were 5 m/s and 9 m/s, respectively. Conversely, at a flow velocity of 5 m/s, the critical depth for severe damage was 3 m. Stress analysis showed that boulder impact induced significant stress concentration, with the peak von Mises stress (*σ*_boulder_) 114.29% higher than that from the slurry (*σ*_slurry_). A damage grading standard and corresponding displacement prediction formulas were proposed based on the inter-story displacement angle. Collectively, these findings provide a theoretical and practical foundation for designing rural masonry structures that are resilient to debris flow disasters.

## 1. Introduction

The Three Gorges Reservoir Area in China features complex geomorphology and significant climate variations, leading to a high frequency of debris flow hazards. In these rural regions, masonry structures remain the predominant construction type due to local economic constraints. However, masonry structures generally exhibit low resistance to impact loads. When subjected to impact from a debris flow containing boulders, these structures are highly vulnerable to localized damage or even global collapse. These structural failures often result in severe infrastructure damage and significant human casualties [1,2]. This research will provide a scientific basis for designing, reinforcing, and protecting rural masonry structures in areas with high debris flow hazards, thereby enhancing resilience in mountainous regions. Thus, this study aims to develop a validated Fluid-Structure Interaction (FSI) model for simulating debris flow impact on masonry structures, analyze the dynamic response and failure mechanisms under varying flow conditions, establish critical damage thresholds, and propose practical displacement prediction models and damage classification standards.

The dynamic response and failure mechanisms of masonry structures under debris flow impact represent a critical research frontier in geohazard mitigation. Consequently, a growing body of recent studies has begun to address this topic [3–6]. However, existing research primarily focuses on the global scouring and burial effects of debris flow slurry [7]. It provides insufficient quantification of stress concentration induced by boulder impacts and lacks an understanding of the synergistic disaster mechanisms among boulders, slurry, and structures [8,9]. Research on boulder impact failure modes and mechanisms remains particularly limited for masonry, given its inherent heterogeneity and brittle behavior. The presence of boulders fundamentally alters the impact characteristics of debris flows [10,11]. Moreover, stochastic variations in boulder morphology, impact position, and density are recognized as significant factors influencing the resulting failure modes. Critically, the influence of these stochastic factors on key failure thresholds such as inter-story displacement angle requires rigorous quantitative investigation. Furthermore, current models for the interaction between debris flows and structures inadequately simulate the key physical processes involved [12]. While the dynamic response of reinforced concrete frames to boulder impacts has been well studied [13–15], the heterogeneous material properties and brittle failure characteristics of masonry lead to localized failure mechanisms that are fundamentally distinct from those of frame structures. Consequently, findings from RC frame structure studies cannot be directly extrapolated to masonry structures.

The Fluid-Structure Interaction (FSI) method provides an effective approach for simulating the impact of debris flows on structures, particularly when seeking a balance between computational efficiency and simulation accuracy. While multiphase flow models enable detailed simulation of interactions among boulders, slurry, and structures [15], their substantial computational demand limits their applicability in disaster emergency assessment. Although Iverson’s physical model for debris flows [16] established the theoretical foundation for such multiphase simulations, their computational complexity hinders practical engineering implementation, particularly for emergency assessment. Consequently, the development of efficient yet accurate models has become a key research priority. Liu et al. [5] applied a monophasic Bingham model to simulate debris flows and demonstrated its efficacy in predicting impact force distributions. In another study, Huang et al. [17] employed coupled simulations to validate the reliability of the FSI method for predicting building sheltering effects. However, existing models exhibit three key limitations: they oversimplify masonry material nonlinearity and progressive damage mechanisms, inadequately represent mortar-block interface failure criteria, and neglect the stochastic nature of boulder impacts [10]. This study adopts the finite element method to implement FSI and a monophasic Bingham model to balance precision and computational efficiency. Furthermore, this approach also provides a foundation for subsequent investigations that may employ machine learning and artificial intelligence to advance more complex multiphase flow models for broader engineering applications.

This study identifies three key limitations in existing research: First, the understanding of localized damage mechanisms induced by boulders remains limited, as existing research has failed to quantify the associated stress concentration effects and damage thresholds in masonry. Second, existing FSI models for interactions between debris flows and masonry structures oversimplify the physical processes, such as material nonlinearity, progressive damage, and interface failure. Third, the engineering applicability of these FSI models is severely constrained by their high computational cost. Therefore, this study developed an advanced FSI model in ANSYS Workbench, validated against experimental data, to simulate the dynamic response of masonry structures impacted by debris flow containing boulders. This provides a foundation for subsequent analysis. Our study systematically analyzed the dynamic response and failure mechanisms of the structures. Specifically, we analyzed the time history characteristics of impact forces from debris flows containing boulders and established their correlations with flow velocity and depth. Furthermore, the coupled disaster mechanisms of boulder and slurry impacts were investigated. A damage classification system for masonry structures under debris flows was established based on inter-story displacement angle thresholds. This study identified critical thresholds for structural resistance through systematic analysis of various flow velocities and depths. These results provide a theoretical basis for key parameters in debris flow risk assessment and a foundation for developing protective strategies to enhance the resilience of rural masonry structures against debris flows.

## 2. Materials and methods

This section describes the development and validation of the Fluid-Structure Interaction (FSI) model, including the constitutive models for debris flow and masonry, numerical setup, and parametric analysis procedures.

### 2.1 Fluid-structure interaction (FSI) modeling

#### 2.1.1 Overview of the ANSYS FSI.

Fluid-Structure Interaction (FSI) is a computational methodology to simulate coupled phenomena between fluid flows and deformable structures. This approach enables accurate simulation of fluid-structure system’s dynamic response by establishing coupling between the governing equations of fluid flow and solid mechanics. The core of the methodology lies in the simultaneous solution of the governing equations for fluid and structural dynamics. An iterative algorithm facilitates data exchange at the interface, capturing both the effects of fluid pressure and velocity fields on structural stress and deformation, and the feedback of structural deformations on the fluid field distribution. The method enables systematic elucidation of complex Fluid-Structure Interaction mechanisms and establishes a theoretical foundation for safety design and protection analysis of engineering structures [21].

This study employed the Fluid-Structure Interaction (FSI) capability in ANSYS Workbench to simulate the complex multiphysics interactions between fluid and structural domains using the System Coupler. The governing equations for the fluid domain were solved using the Fluent module, while those for the solid domain were handled by the Transient Structural module. In this bidirectional coupling scheme, the Fluent module first transferred the fluid pressure and shear forces at the interface to the Transient Structural module as applied loads. The Transient Structural module then calculated the resulting structural deformation and fed the displacement data back to Fluent, where the fluid mesh was updated accordingly. This iterative process was carried out at each time step to capture the transient response of the coupled fluid-structure system.

#### 2.1.2 Bingham rheological model.

Debris flows are a distinct type of non-Newtonian fluid. The Bingham model is widely used to characterize the relationship between shear stress and strain rate in slurry mixtures [18]. This constitutive model has shown good agreement with experimental data in debris flow simulations [11,19]. Compared to complex multiphase flow models [18], the single-phase Bingham model maintains accuracy while reducing computational complexity, making it suitable for rapid assessment of large-scale debris flow hazards [19]. To optimize computational efficiency, particulate-scale interactions such as sedimentation and erosion are neglected, as this study focuses on the macroscale mechanical response of masonry structures to debris flow impact. Therefore, the simulations employ the Bingham plastic model. Existing research has demonstrated that this model predicts both the peak impact force and spatial distribution pattern of debris flows with errors within acceptable engineering tolerances [16].

#### 2.1.3 Drucker-Prager constitutive model.

Masonry structures exhibit pronounced nonlinear mechanical behavior with distinct tensile and compressive strengths. The Drucker-Prager model [20] was adopted to characterize the elastoplastic behavior of the masonry structures. In this model, yield behavior under complex stress states is defined primarily by the friction angle and cohesion parameters [21]. The material strength parameters for the model were determined according to the specifications in Appendix A of the Code for Design of Masonry Structures GB 50003−2011 [22]. Given the primary focus on macroscale dynamic responses to debris flow impact and the substantial number of elements in the model, a homogenized Drucker-Prager model was employed to balance computational accuracy and efficiency. Future work will investigate mesoscale damage evolution at mortar-block interfaces using a coupled DEM-SPH-FEM multiscale modeling approach [14].

#### 2.1.4 FSI and turbulence modeling.

An FSI model was developed in ANSYS Workbench to simulate debris flow impact on a structure and analyze the resulting macroscale fluid-structure responses [2,13]. Debris flow movement is a highly turbulent process, exhibiting strongly nonlinear and transient dynamics. The RNG k-ε model, which has been validated for accurately predicting the turbulent dynamics of debris flows, was employed for this simulation. Its complete theoretical formulation is provided in reference [23].

To ensure numerical convergence and stability, the residual convergence thresholds were set at 10 ⁻ ⁴ for the fluid continuity, momentum, and energy equations, with a structural displacement tolerance of 10 ⁻ ⁶ [24]. An initial time step of 0.001 s was used in a scheme with adaptive time steps to balance computational efficiency and accuracy in the transient simulation. Numerical results demonstrate that fluid residuals typically decay below 10 ⁻ ⁵ within 100 iterations per time step, thus satisfying the convergence criteria [24].

#### 2.1.5 Constitutive material relationships.

The uniaxial compressive and tensile stress-strain relationships for concrete were modeled following the specifications in Articles C.2.3 and C.2.2 of the Chinese Code for Design of Concrete Structures (GB 50010−2010). A Poisson’s ratio of 0.2 was adopted for the concrete [25]. The reinforcing steel was modeled as an ideal elasto-plastic material, and its stress remained within the elastic range in all analyzed scenarios.

The uniaxial compression formulation for masonry structures was obtained from [21], while the corresponding constitutive model parameters were obtained from [22]. The yield strain (*ε*_y_) was calculated according to the Code for Design of Masonry Structures (GB 50003−2011) [22], and the ultimate compressive strain (*ε*_cu_) was defined as 10 times *ε*_y_ based on Reference [26]. The tensile constitutive relationship and the corresponding ultimate tensile strain values were obtained from [26]. The elastic modulus (E) and a Poisson’s ratio of 0.15 for the masonry were adopted from [22].

#### 2.1.6 Validation of the model.

**Fig 1:**
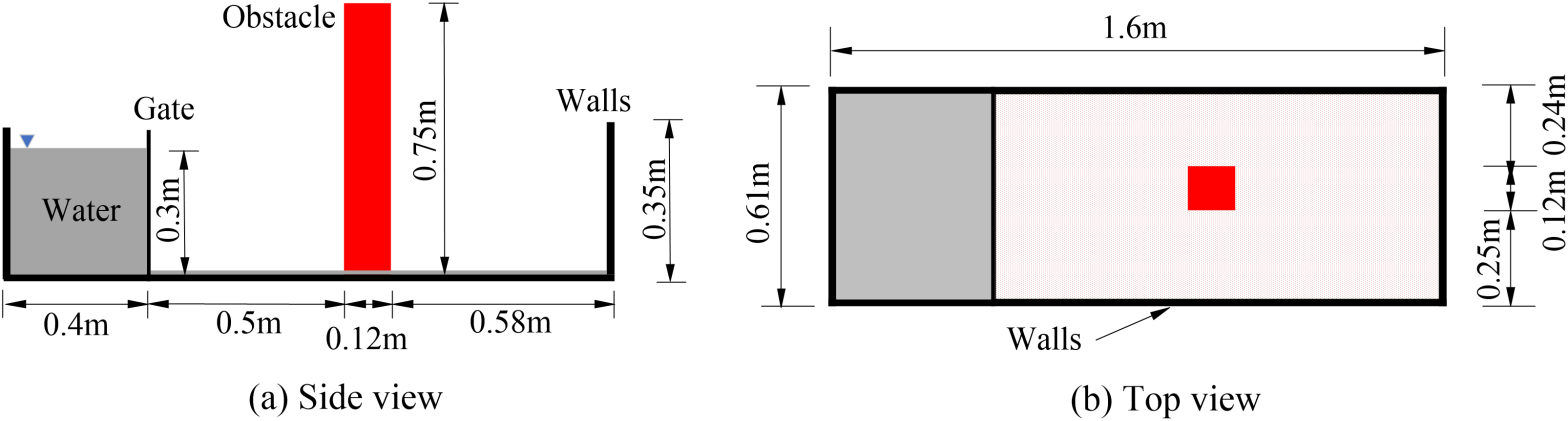
Geometric dimensions of the experimental water impact model adapted from [27].

To validate the model for fluid dynamics and FSI, we reproduced the water impact experiment on a rigid obstacle by Gómez-Gesteira and Robert [27]. A schematic of this experiment is presented in Fig 1. In the numerical model, the water domain, flume tank, and rigid obstacle were meshed with SOLID186 elements (3D 20-node hexahedral solids). The water domain was assigned a 5 mm mesh, whereas the obstacle and flume were assigned a coarser 10 mm mesh. The rigid obstacle was fully constrained at its base to the flume bottom. The load transfer between the fluid and obstacle was applied at the FSI interfaces. The water density was 1000 kg/m^3^, with a dynamic viscosity of 0.001 Pa·s.

**Fig 2:**
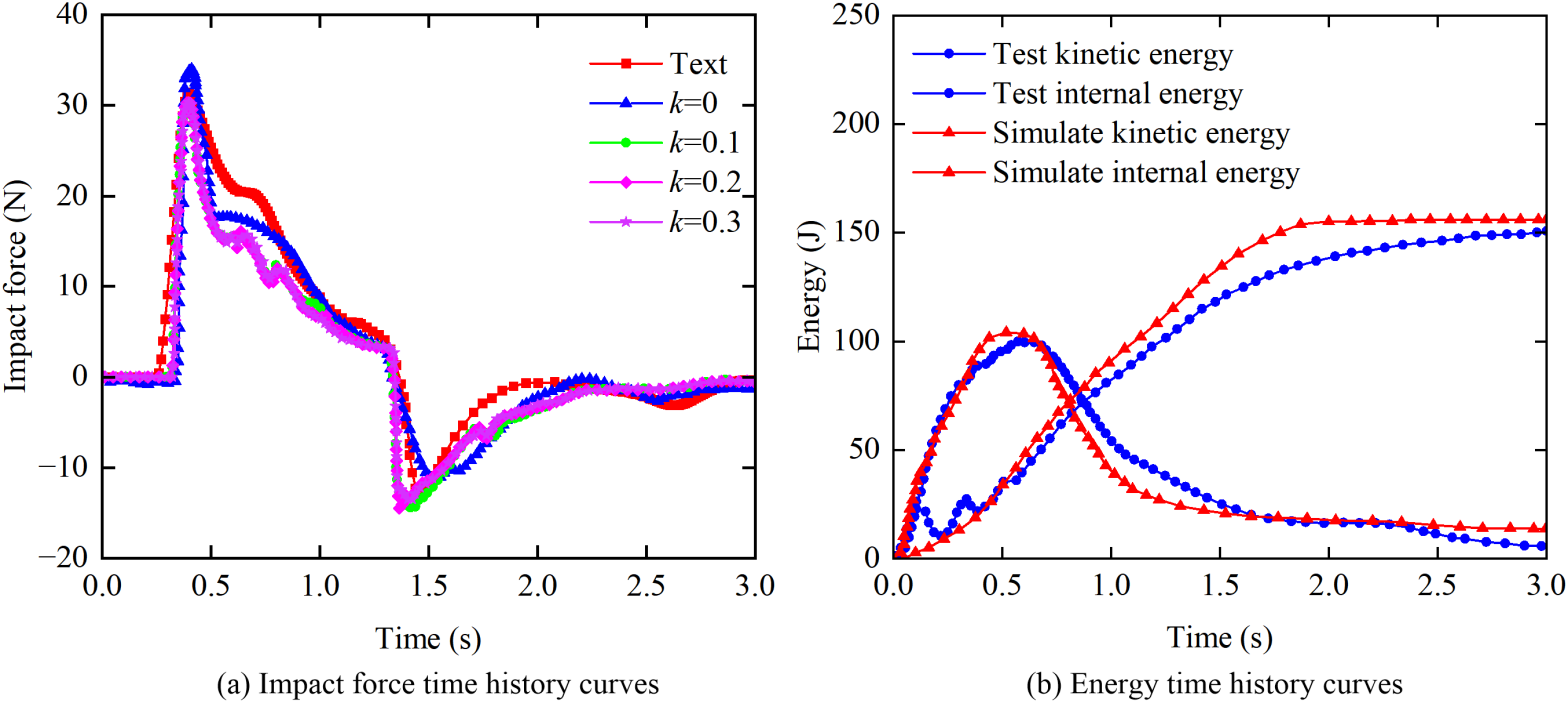
Comparison of impact force and energy between the numerical simulation and the experimental data from Gómez-Gesteira and Robert [26].

Numerical simulations were conducted to evaluate the impact force response for four different values of the fluid-structure sliding friction coefficient (*k*): 0, 0.1, 0.2, and 0.3. Fig 2 compares the time histories of the impact force from the present simulation with the experimental data from [27]. The peak value in these curves corresponds to the impact force on the upstream face of the obstacle, whereas the minimum value represents the force on the downstream side, which is attributed to negative pressure. The results presented in Fig 2 indicate that the sliding friction coefficient has a minimal influence on the impact force, and that the numerical simulation data show excellent agreement with the experimental measurements over the entire time history. The experimentally measured peak impact force was 33.27 N, compared to the simulated peaks of 31.51 N, 30.52 N, 30.58 N, and 30.63 N for *k* values of 0, 0.1, 0.2, and 0.3, respectively. For *k* = 0, the simulated peak differed from the experimental value by 1.76 N, representing a relative error of 5.29%; for *k* = 0.3, the difference was 3.06 N, corresponding to a relative error of 7.94%. All discrepancies were within acceptable engineering tolerances.

Additionally, the kinetic energy and internal energy from the numerical simulations were compared with those from the experiments. Fig 2(b) shows the time history curves of kinetic and internal energy for *k* = 0, demonstrating that the simulation results closely match the experimental data. The simulated peak internal and kinetic energies were 155.95 J and 104.1 J, respectively. These values correspond to relative differences of 3.9% and 4.0% compared to the experimental measurements of 150 J and 99.81 J.

A numerical model was developed using ANSYS Workbench to simulate water impact processes on a rigid obstacle structure. The simulation results agree well with experimental data, which demonstrates the reliability of the employed Fluid-Structure Interaction (FSI) algorithm. Furthermore, this work provides a basis for extending the present numerical method to simulate the dynamic response of masonry structures impacted by debris flow containing boulders.

### 2.2 Failure modes and numerical modeling of masonry structures under impact from debris flows

#### 2.2.1 The july 2023 debris flow event in Wanzhou, China.

**Fig 3:**
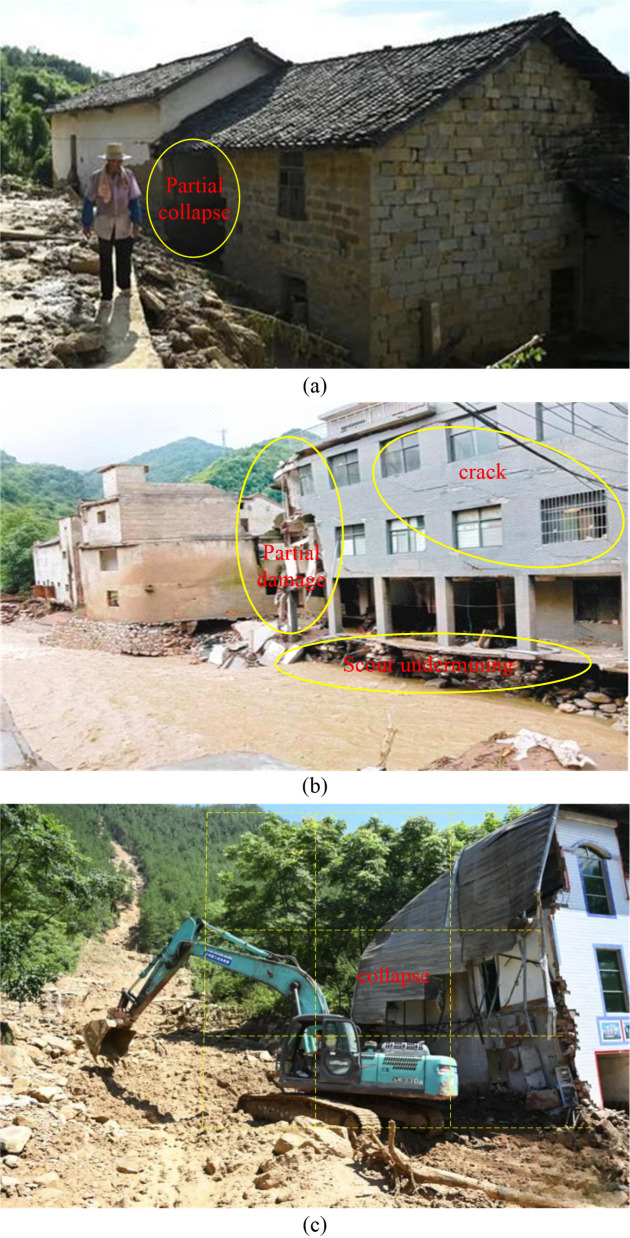
Building damage caused by debris flow in Changtan town, Wanzhou, Chongqing, China, July 2023.

In July 2023, extreme rainfall triggered cascading landslides and debris flows in Changtan, Wanzhou, causing extensive damage to rural masonry structures. As shown in Fig 3, the characteristic failure modes included: (a) Foundation scour: debris flow containing boulders eroded the foundations, resulting in differential settlement exceeding 120 mm. (b) Shear failure: direct impact from boulders caused partial collapse of the ground-floor load-bearing walls. (c) Global overturning: total collapse occurred when the lateral thrust of the debris flow exceeded the structure’s overturning resistance moment. These failure modes documented during field investigations provided critical benchmarks for validating the subsequent numerical model’s predictive capability for structural damage.

#### 2.2.2 Description of the masonry structure.

**Fig 4:**
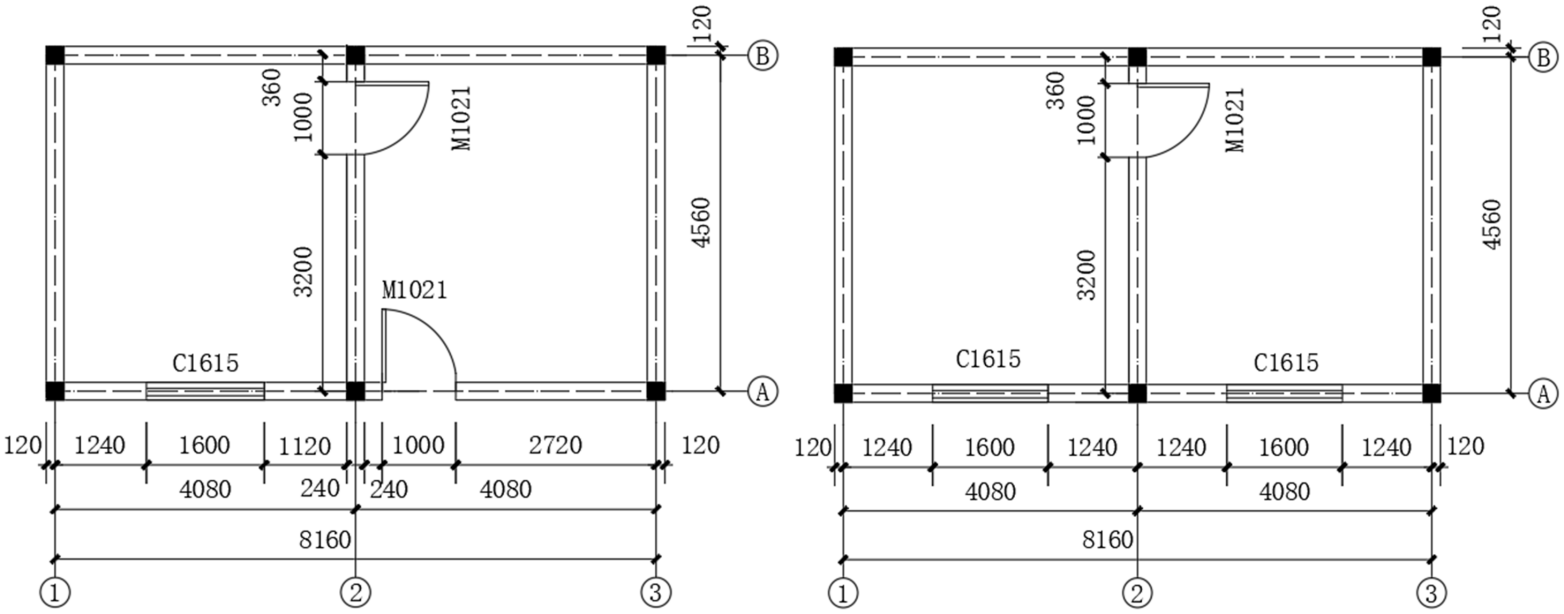
Architectural floor plan of the masonry structure.

Following field investigations in the debris flow disaster zones of Changtan Town, Wanzhou District, Chongqing, in July 2023, this study used numerical simulations to investigate the dynamic response of confined masonry structures impacted by debris flow containing boulders. The analysis focused on a two-story masonry structure (Fig 4). The building was constructed in 2002 and founded on bedrock using strip stone footings. Each story had a height of 3.2 m, yielding a total building height of 6.4 m. The load-bearing walls were 240 mm thick and constructed of MU10 grade fired clay bricks with M7.5 mortar. The principal structural members included confining columns with 240 mm × 240 mm cross-sections, reinforced with 4 Φ12 HRB335 longitudinal bars and ϕ6 transverse ties at 200 mm spacing. Bond beams had a cross-section of 240 mm × 180 mm and were reinforced with 4 Φ12 HRB335 longitudinal bars and ϕ6 ties at 200 mm spacing. The cast-in-place concrete slabs were 100 mm thick, made of C25 concrete, and reinforced with a ϕ6 mesh spaced at 100 mm in both directions. The materials and mechanical properties of the masonry structure are summarized in Table 1; these properties comply with Chinese national standards GB 50003−2011 [22] and GB 50010−2010 [25]. According to Chinese Code GB 50009−2012, the characteristic values of the service loads were assigned as follows: permanent loads of 2.5 kN/m^2^ for both floors and roof, live loads of 2.0 kN/m^2^ for floors, and live loads of 0.5 kN/m^2^ for the roof.

**Table 1:** Materials and mechanical properties [22,25].

Material	Strength Class	Density (kg/m^3^)	Young’s modulus (MPa)	Poisson’s ratio	Design Compressive Strength (MPa)
**Masonry wall**	MU10M7.5	1700	2700	0.15	1.69
**Transverse reinforcement**	HPB235	7850	2.1 × 10^5^	0.3	210
**Longitudinal reinforcement**	HRB335	7850	2 × 10^5^	0.3	300
**Concrete**	C25	2500	2.8 × 10^4^	0.2	11.9
**Stone**	MU80	2700	2 × 10^4^	0.25	——

#### 2.2.3 Numerical modeling.

SOLID186 hexahedral elements were used to discretize both the debris flow and the structural members. The interfaces between masonry walls and other structural components were modeled as tied constraints. The reinforcing steel was modeled using beam elements and embedded within the corresponding concrete members [28]. A minimum of two elements were maintained through the thickness of all members to prevent numerical instabilities and ensure solution accuracy.

The masonry walls, ring beams, and structural columns had a uniform thickness of 240 mm. In the finite element models, the element mesh size for these components was limited to 120 mm. To evaluate the influence of mesh density on the structural modal analysis results, a mesh sensitivity analysis was performed using element sizes of 120 mm and 60 mm for these components. The floor slab was modeled with a uniform mesh of 50 mm.

The first six natural frequencies for the two mesh configurations are summarized in Table 2. Mesh refinement from 120 mm to 60 mm increased the computation time from 583 seconds to 1100 seconds, yet the difference in the first natural frequency between the models was merely 0.12%. The negligible differences in dynamic characteristics indicated that the solution converged with respect to mesh density, and further refinement would offer limited improvement in accuracy. Consequently, a mesh size of 120 mm was adopted for these structural components to balance computational efficiency with accuracy. Additionally, the boulder and debris flow slurry were meshed at 100 mm and 120 mm, respectively, resulting in a total of 606,936 elements. This mesh sensitivity analysis followed established practice [11] and confirmed that the selected mesh sizes ensured computational accuracy.

**Table 2:** Influence of mesh size on computational cost and modal frequencies of a masonry structure.

Mesh size	Computation time	Analytical factors	First mode	Second mode	Third mode	Fourth mode	Fifth mode	Sixth mode
**120mm**	583 seconds	Frequency	16.662	17.291	23.769	23.81	25.911	27.086
**60mm**	1100 seconds	Frequency	16.642	17.242	23.74	23.79	25.89	27.04
**Duration difference**	188.68%	Frequency difference	0.12%	0.28%	0.12%	0.08%	0.08%	0.17%

**Fig 5:**
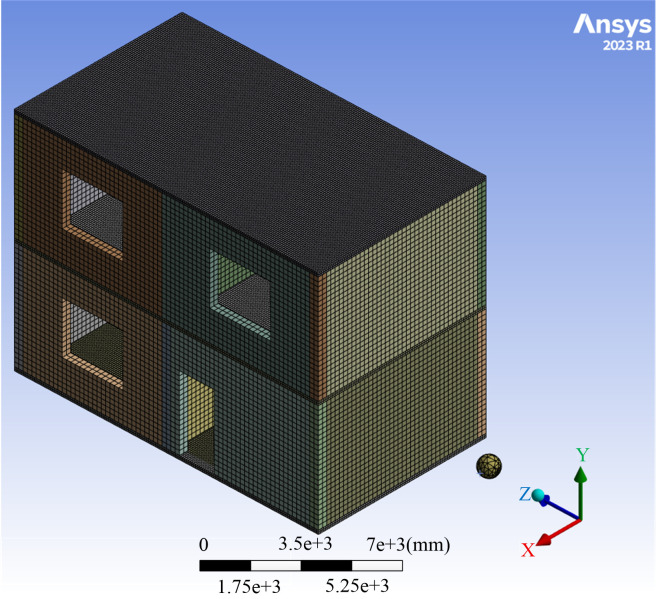
Numerical model.

The model was based on several fundamental assumptions. The masonry structure was fully fixed at its base. The boulders were idealized as rigid bodies that could only translate in the debris flow direction, and their rotational degrees of freedom were neglected. The interaction between the debris flow slurry and the masonry was simulated using a surface-to-surface contact algorithm, with a friction coefficient of 0.3 defined at the interfaces [10]. Both the slurry and boulders were assigned the same initial velocity in the impact direction [29,30]. The total simulation duration was set to 3 s. The final numerical model is depicted in Fig 5.

### 2.3 Working condition parameters

Field investigations demonstrated that the impact damage to masonry structures from debris flows stemmed primarily from the combined action of slurry and entrained boulders [30]. This combined action constituted the predominant failure mechanism for rural masonry structures. To investigate these failure mechanisms, a Fluid-Structure Interaction (FSI) model was developed to simulate the impact of debris flows containing boulders on masonry structures.

The numerical model was calibrated with data from a typical debris flow event monitored at the Dongchuan Debris Flow Observation and Research Station (DFSORS) of the Chinese Academy of Sciences (CAS). This event, which occurred in Jiangjia Gully on 2 August 2023, provided critical measurements including a bulk density of 2.09 × 10³ kg/m³, a peak velocity of 6.8 m/s, and a flow depth ranging from 0.5 to 2.3 m [31]. The computational domain had dimensions of 20.4 m in length, 14.4 m in width, and 1.5 m in height [2].

Previous investigations have shown that boulders in debris flows are typically concentrated within specific depth zones. Yang et al. [11] reported that boulders are predominantly concentrated within 0.4 to 0.6 times the flow depth. Wei [10] found that boulder impact heights in dilute debris flows are typically clustered around one third of the flow depth. In the case of viscous debris flows, He et al. [32] found that the most severe damage occurs when boulders are located at the middle of the flow depth. Based on these findings, the boulder distribution depth in this study was conservatively defined as two thirds of the flow depth to account for potential impacts on the upper portions of the structure.

**Fig 6:**
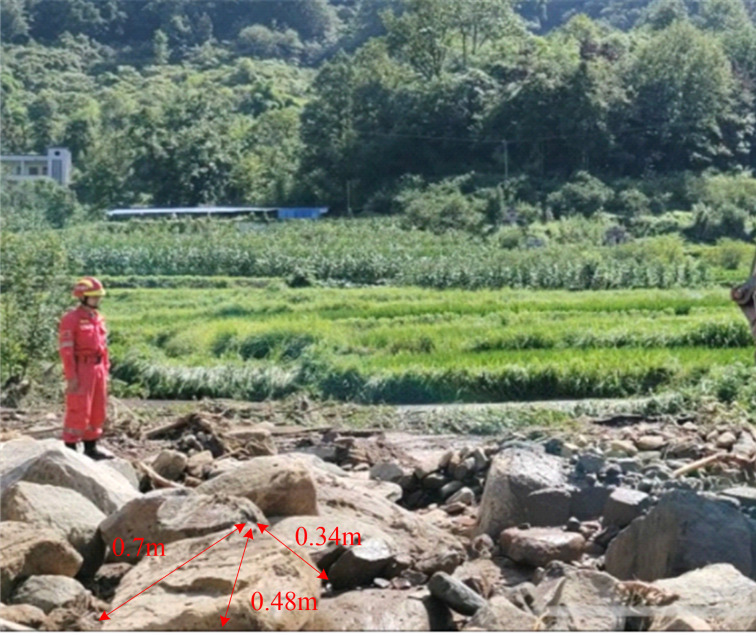
Boulder size measured in the field.

Conventional approaches typically model the boulder impact force as a static concentrated load based on established theoretical formulas [33], which neglects the dynamic nature of the impact process. To simulate boulder impacts, the irregular boulders were idealized as spheres with a diameter of 0.5 m. This simplification was based on field measurements of boulder size (Fig 6), and the equivalent volume method was applied to determine the spherical diameter [30]. Although this approach preserves momentum conservation [33], it neglects the effects of stress concentration at angular boulder contacts. The FSI model was employed to simulate debris flow interactions with masonry structures containing these equivalent boulders. This model enables dynamic reconstruction of the entire impact process and detailed time history analysis of the impact force. This spherical simplification may underestimate localized stress by up to 15% compared to angular rocks [33]. This limitation will be addressed in future work using the Discrete Element Method (DEM).

A parametric study was performed to investigate the influence of debris flow velocity and depth on the impact force and dynamic response of masonry structures. The study was based on the 2 August 2023 Jiangjia Gully event, as documented by the DFSORS of the CAS. The impact angle was fixed at 90° for all cases. Table 3 summarizes the simulation cases with varying velocities at a flow depth of 1.5 m, and Table 4 details those with varying flow depths at a velocity of 5 m/s.

**Table 3:** Parametric study cases for flow velocity variations.

V (m/s)	3	4	5	6	7	8
**Cases**	A90V3F0.19D1.5	A90V4F0.35D1.5	A90V5F0.53D1.5	A90V6F0.77D1.5	A90V7F1.0D1.5	A90V8F1.36D1.5

*A*, Impact angle; *V,* Flow velocity; *F*, Froude number; *D*, Flow depth.

**Table 4:** Parametric study cases for flow depth variations.

D (m)	1	1.5	2	2.5	3
**Cases**	A90V5F0.53D1	A90V5F0.53D1.5	A90V5F0.53D2	A90V5F0.53D2.5	A90V5F0.53D3

## 3. Numerical simulation results

### 3.1 Impact force

#### 3.1.1 Dynamic process analysis of debris flow impact.

**Fig 7:**
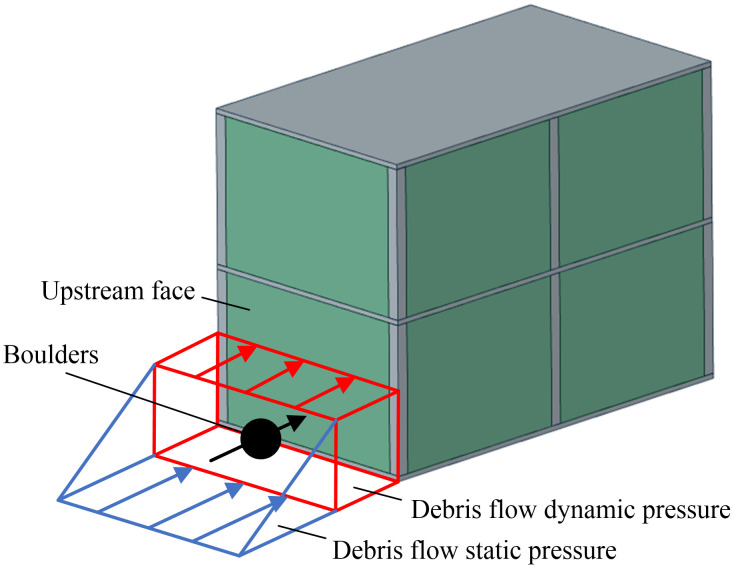
Schematic of debris flow impact on a masonry structure.

Fig 7 schematically illustrates the impact process of debris flow containing boulders on a masonry structure. The flow front is high-velocity slurry. The main flow region represents the core zone of boulder movement, with boulders traveling at a mean depth of 1.0 m. The wake zone is dominated by turbulent flow.

**Fig 8:**
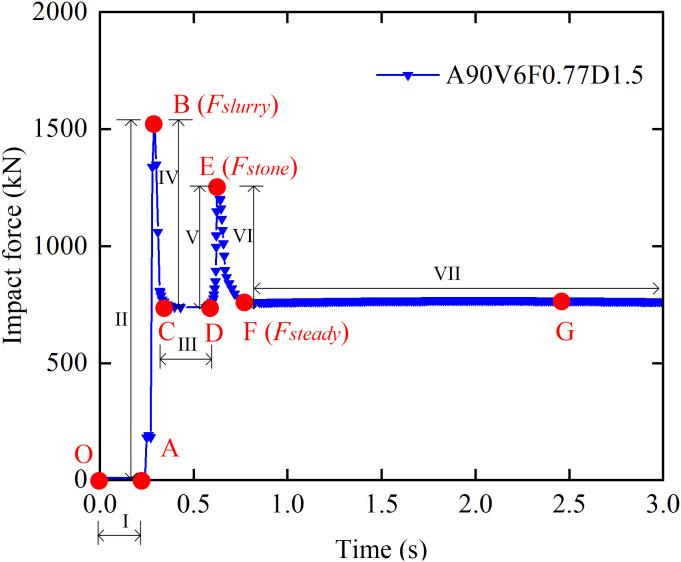
Time history of the impact force for condition A90V6F0.77D1.5.

Fig 8 illustrates the time history of the impact force on a masonry structure from a debris flow containing boulders, corresponding to simulation condition A90V6F0.77D1.5, which represents an impact angle of 90°, a flow velocity of 6 m/s, a Froude number of 0.77, and a flow depth of 1.5 m. The curve shows seven distinct phases characterized by a dual-peak pattern. During Phase OA, the debris flow approaches the structure without making contact, and the impact force remains zero. Phase AB involves the impact of the frontal slurry, which rapidly increases the force to the first peak, *F*_slurry_ [15]. In Phase BC, the kinetic energy of the slurry is converted into elastic-plastic strain energy within the masonry structure, resulting in force attenuation that stabilizes at a steady value, *F*_steady_. Phase CD is a brief stable phase where the force remains at *F*_steady_ immediately before the boulder impact. In Phase DE, the impact of a boulder at *t* = 0.62 s triggers the second peak force, *F*_boulder_. The second peak exhibits a phase lag of ∆*t* = 0.32 s relative to *F*_slurry_, resulting from the dissipation of boulder energy through viscous drag within the slurry [34]. During Phase EF, the boulder’s kinetic energy is dissipated through structural plastic deformation, causing the force to decay again to *F*_steady_. In Phase FG, a low velocity wake zone develops downstream, inducing a ‌sheltering effect‌ [35] that maintains the force at *F*_steady_.

**Fig 9:**
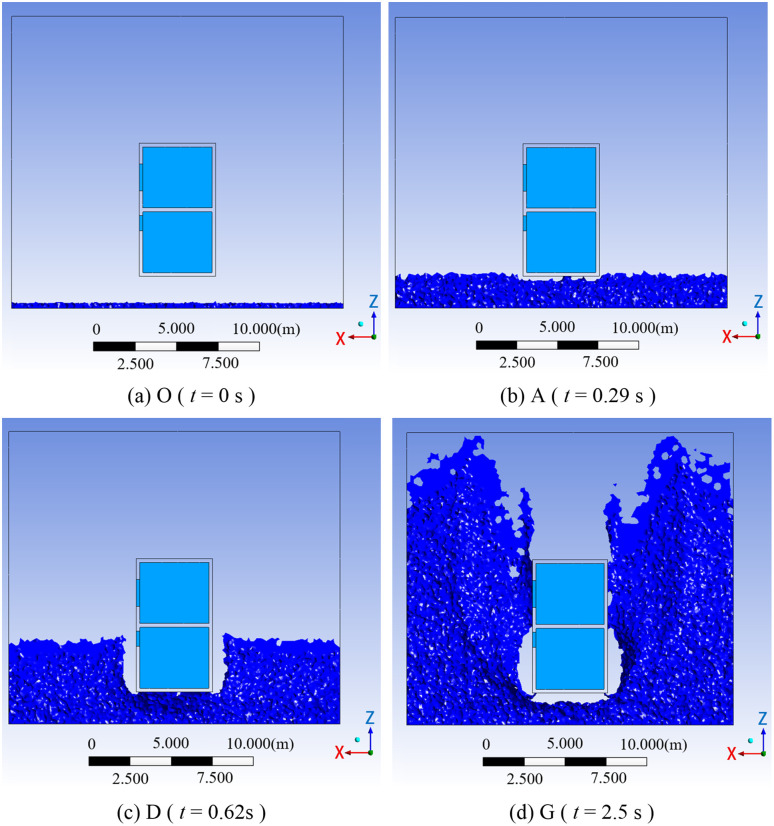
Spatiotemporal evolution of a debris flow containing boulders impacting a masonry structure.

Figs 9(a) to 9(d) illustrate the spatiotemporal evolution of the debris flow impact process presented in Fig 8, at the specific time points O (*t* = 0 s), A (*t* = 0.29 s), D (*t* = 0.62 s), and G (*t* = 2.50 s). Specifically, Fig 9(a) shows the initial state before the interaction between the debris flow and the structure at *t* = 0 s. Fig 9(b) shows the initial contact of the flow front with the structure at *t* = 0.29 s. Fig 9(c) shows the impact of a boulder on the structure at *t* = 0.62 s. Finally, Fig 9(d) shows the stabilized state of the flow at *t* = 2.50 s.

#### 3.1.2 Effect of flow velocity on impact force.

**Fig 10:**
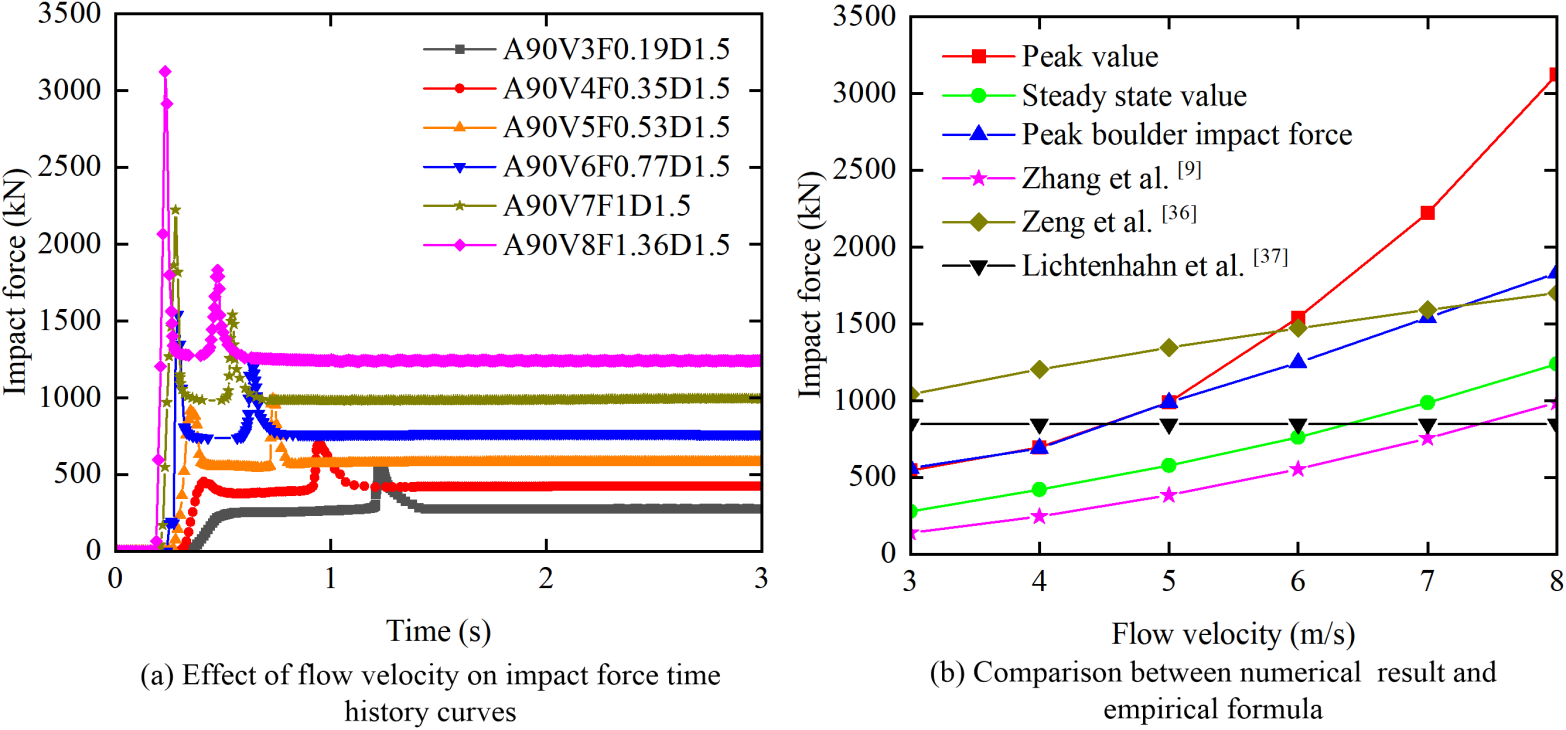
Effect of flow velocity on the impact force.

Fig 10(a) shows the influence of flow velocity on the evolution of the impact force time history. With the exception of case A90V3F0.19D1.5, all investigated cases exhibit a characteristic seven-stage evolution pattern with a bimodal force distribution. Both peak and steady state impact forces increase monotonically with flow velocity. A critical flow velocity of approximately 5 m/s governs the transition of the dominant impact force peak. When *V* ≤ 5 m/s, the secondary peak (*F*_boulder_) exceeds the initial slurry peak (*F*_slurry_), indicating that boulder kinetic energy is the prevailing factor. Conversely, when *V* > 5 m/s, *F*_slurry_ exceeds *F*_boulder_, indicating that the inertial force of the slurry becomes predominant. In the velocity range of 3–4 m/s, *F*_slurry_ approaches *F*_steady_. An increase in flow velocity significantly reduces the response time of the impact system. The phase lag between peaks (∆*t*) decreases from 0.72 s a*t V =* 3 m/s to 0.24 s at *V =* 8 m/s. The stabilization time decreases from 1.40 s at *V =* 3 m/s to 0.63 s at *V =* 8 m/s.

Fig 10 (b) compares the simulated impact forces with the theoretical predictions at flow velocities ranging from 3 to 8 m/s. The corresponding quantitative data are summarized in Table 5. The results identify a critical velocity of 5 m/s for the shift in dominance between the peak slurry impact force (*F*_slurry_) and the peak boulder impact force (*F*_boulder_), which establishes this velocity as a key control parameter for protective engineering design. Furthermore, this study reveals a significant limitation of existing theoretical models: they underestimate the peak impact forces by a factor of 1.1 to 3.0 when the flow velocity exceeds 5 m/s. This deviation is attributed to the nonlinear pressure distribution along the structure’s height. Numerical results reveal a 170% increase in pressure at the base of the structure relative to the top. Based on these findings, future research should focus on developing high-fidelity Fluid-Structure Interaction models for debris flow and masonry structure systems that incorporate nonlinear pressure distribution effects to more accurately represent actual loading mechanisms.

**Table 5:** Comparison between simulated and theoretical impact forces at flow velocities ranging from 3 to 8 m/s.

Comparative parameters	Numerical regularities	Comparison with the theoretical formulas
**Peak value**	The peak force increased by a factor of 4.7, from 546 kN at 3 m/s to 3 123 kN at 8 m/s.	When *V* ≤ 5 m/s, the measured peak forces are consistent with the theoretical predictions of Zhang et al. [9] and Zeng et al. [36]. However, When *V* > 5 m/s, the peak forces exceed the predictions of Zhang et al. [9], Zeng et al. [36], and Lichtenham et al. [37].
**Steady state value**	The steady state force increased by a factor of 4.46, from 278 kN at *V* = 3 m/s to 1 240 kN at *V* = 8 m/s.	The steady state values are 23% higher than the predictions of Zhang et al. [9] but 27% to 73% lower than those of Zeng et al. [36]. Furthermore, these values are consistently higher than those of Lichtenham et al. [37] when the flow velocities exceed 5 m/s.
**Peak boulder impact force**	At *V* = 8 m/s, the force reaches 1 832 kN, which accounts for 59% of the peak slurry force.	When the flow velocity exceeds 5 m/s, the peak boulder impact force significantly exceeds that predicted by Lichtenham et al. [37].

#### 3.1.3 Effect of flow depth on impact force.

**Fig 11:**
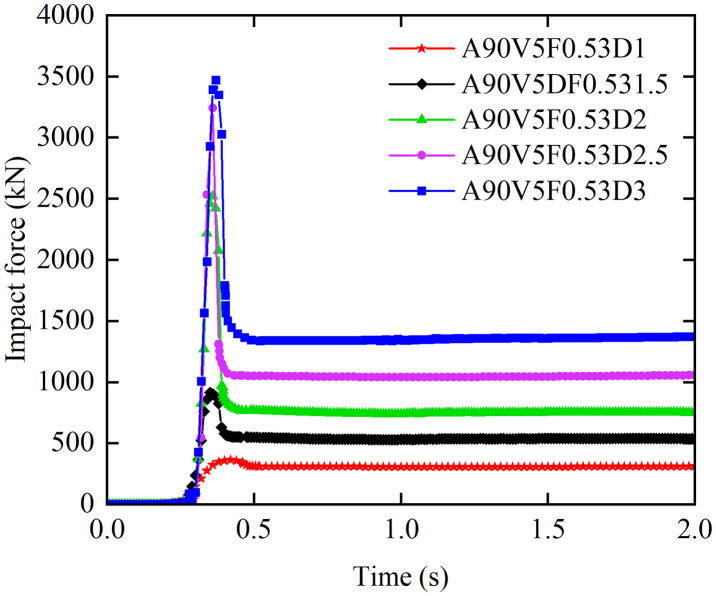
Effect of flow depth on the impact force time history curves.

Fig 11 illustrates the influence of flow depth on the temporal evolution of debris flow impact forces. When boulder effects are neglected, the impact force time history curves exhibit a profile with four phases and a single peak at flow depths of 1.5 m or greater. The stages are defined as follows: Phase I (Pre-impact): The impact force remains at a baseline level. Phase II (Peak formation): The impact force increases abruptly from zero to a peak value, *F*_peak_, which increases nonlinearly with flow depth. Phase III (Attenuation): The impact force decreases by 40–60% from *F*_peak_ to a steady state value, *F*_steady_, with the attenuation rate increasing with flow depth. Phase IV (Steady state): The impact force stabilizes at a steady state value, *F*_steady_, which increases monotonically with flow depth. At a flow depth of 1 m, Phases II and III merge, and no distinct attenuation phase is observed. As the ‌flow depth‌ increases from ‌1 m to 3 m‌, the ‌peak impact force *F*_peak_ increases by a factor of 9.59, and the steady state value *F*_steady_ increases by a factor of 4.77. In summary, the debris flow mass, kinetic energy, and impact force significantly increase with flow depth. Furthermore, increased flow depths substantially elevates the basal pressure on masonry structures, thereby elevating the risk of foundation erosion.

### 3.2 Von Mises stress

#### 3.2.1 Effect of flow velocity on von Mises stress.

**Fig 12:**
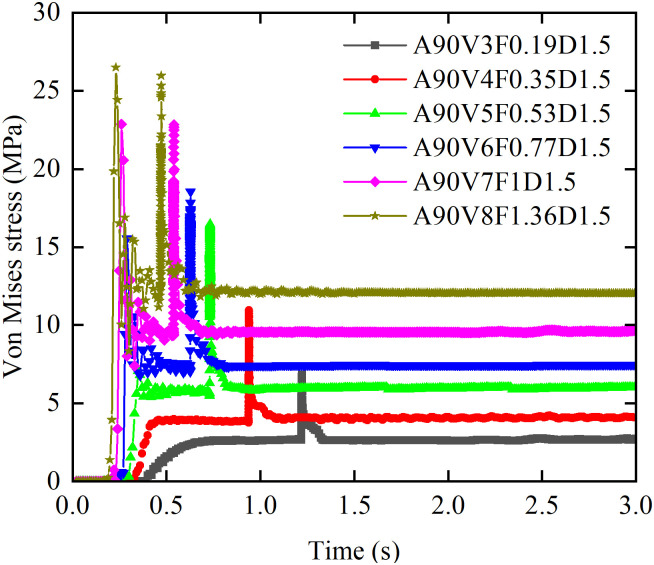
Effect of flow velocity on von Mises stress time history curves.

Fig 12 illustrates the effect of flow velocity on the evolution of the von Mises stress time history curves. At flow velocities of 5 m/s or greater, the von Mises stress time history curves closely mirror those of the impact force, exhibiting the same seven phases, bimodal evolution pattern. At flow velocities of 7 m/s or greater, the primary peak (*σ*_slurry_), induced by slurry impact, exceeds the subsequent peak (*σ*_boulder_), caused by boulder impact. In the flow velocity range from 3 m/s to 6 m/s, the relationship reverses, with *σ*_slurry_ falling below *σ*_boulder_. Furthermore, at flow velocities of 4 m/s or lower, only a single peak (*σ*_boulder_) is observed. The values of *σ*_peak_, *σ*_steady_, and *σ*_boulder_ all exhibit a positive correlation with flow velocity. As the velocity increases from 3 to 8 m/s, all stress parameters are significantly amplified: *σ*_peak_ increases by a factor of 2.9, corresponding to an absolute rise of 19.65 MPa; *σ*_steady_ increases by a factor of 3.65, corresponding to an absolute rise of 9.5 MPa; and *σ*_boulder_ increases by a factor of 2.8, corresponding to an absolute rise of 19.02 MPa.

**Fig 13:**
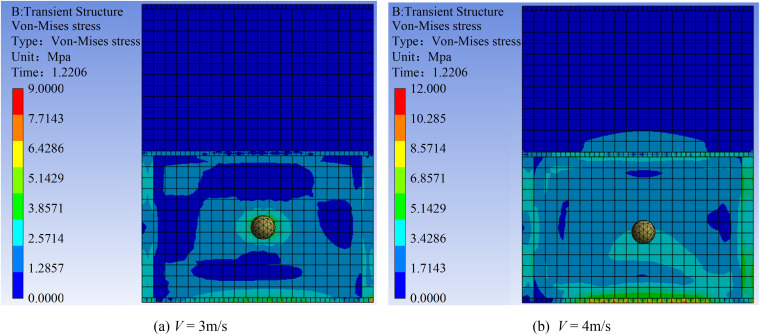
Contours of von Mises stress in the masonry structure at flow velocities of 3 m/s and 4 m/s.

Fig 13 demonstrates the effect of flow velocity on von Mises stress distribution. The stress zone generated by boulder impact at 3 m/s is significantly smaller than that at 4 m/s. By imparting greater kinetic energy upon impact, higher flow velocities generate a larger area of high von Mises stress. At 4 m/s, the stress around the impact zone exhibits a pronounced gradient distribution. The contour plot reveals three distinct zones: blue, indicating low stress; green, indicating medium stress; and yellow, indicating areas of high stress concentration. The maximum von Mises stress (8.57 MPa) occurs within the yellow zone at the structure’s base. This peak value is 4.07 times the compressive strength design value of the masonry, indicating a potential risk of localized crushing.

#### 3.2.2 Effect of flow velocity on maximum principal stress.

**Fig 14:**
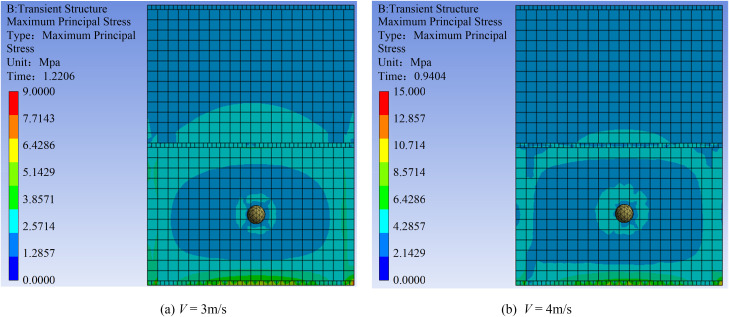
Contours of maximum principal stress in the masonry structure at flow velocities of 3 m/s and 4 m/s.

**Fig 15:**
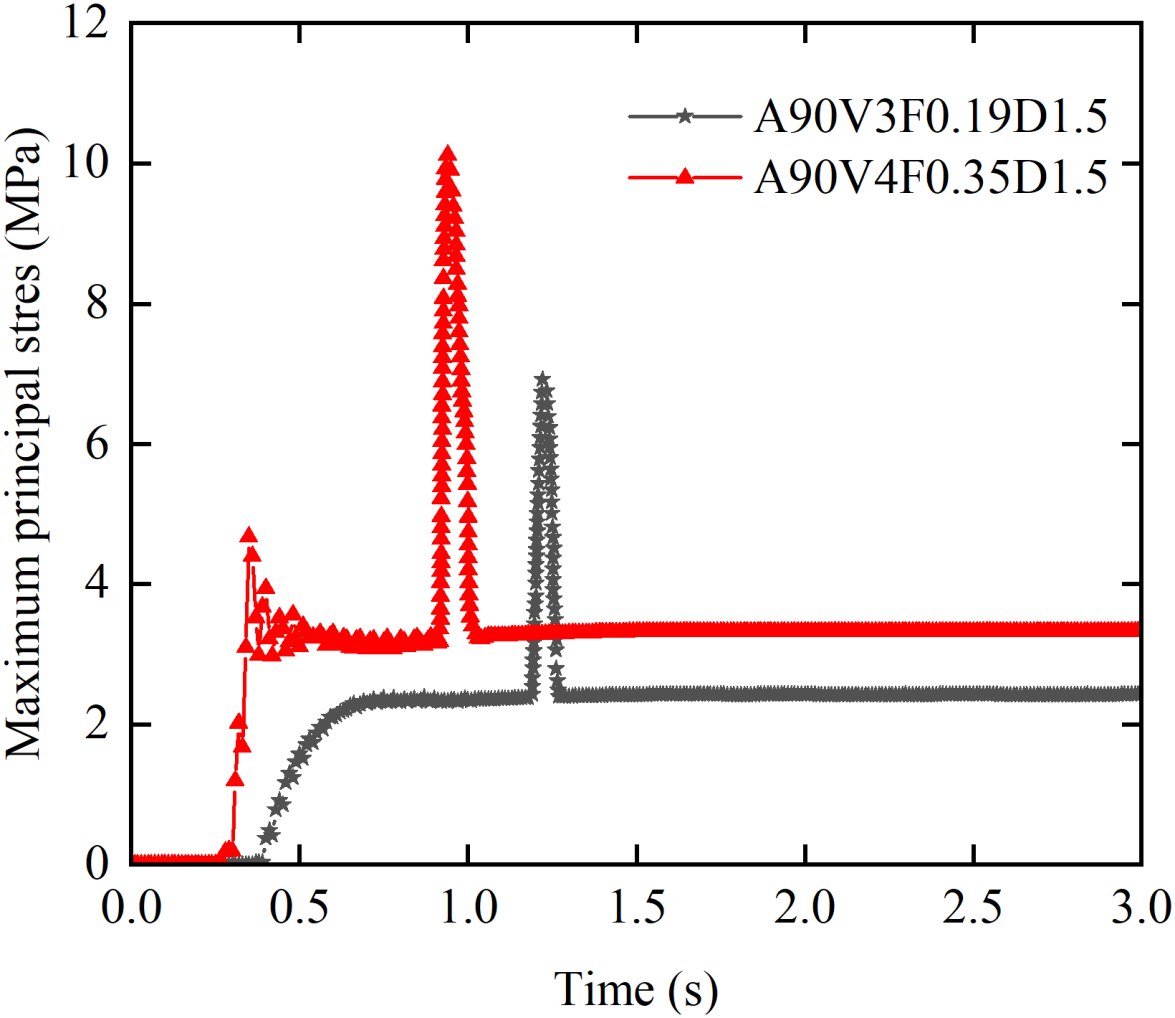
Effect of flow velocity on maximum principal stress time history curves.

Fig 14 illustrates the mechanism by which flow velocity affects the maximum principal stress. The stress zone generated by boulder impact at 3 m/s is 58% smaller than that at 4 m/s. At 4 m/s, the stress around the impact zone exhibits a multilevel gradient distribution. The contours define three distinct regions: a blue region of medium stress; a green region of high stress; and a yellow region of the highest stress concentration. Fig 15 shows that the peak stress increases by 54.8% as the flow velocity rises from 3 to 4 m/s. Within this yellow region, the peak stress reaches 10.71 MPa. This value is 5.34 times the compressive strength design value of the masonry, suggesting that compressive failure occurs.

#### 3.2.3 Effect of flow depth on von Mises stress.

**Fig 16:**
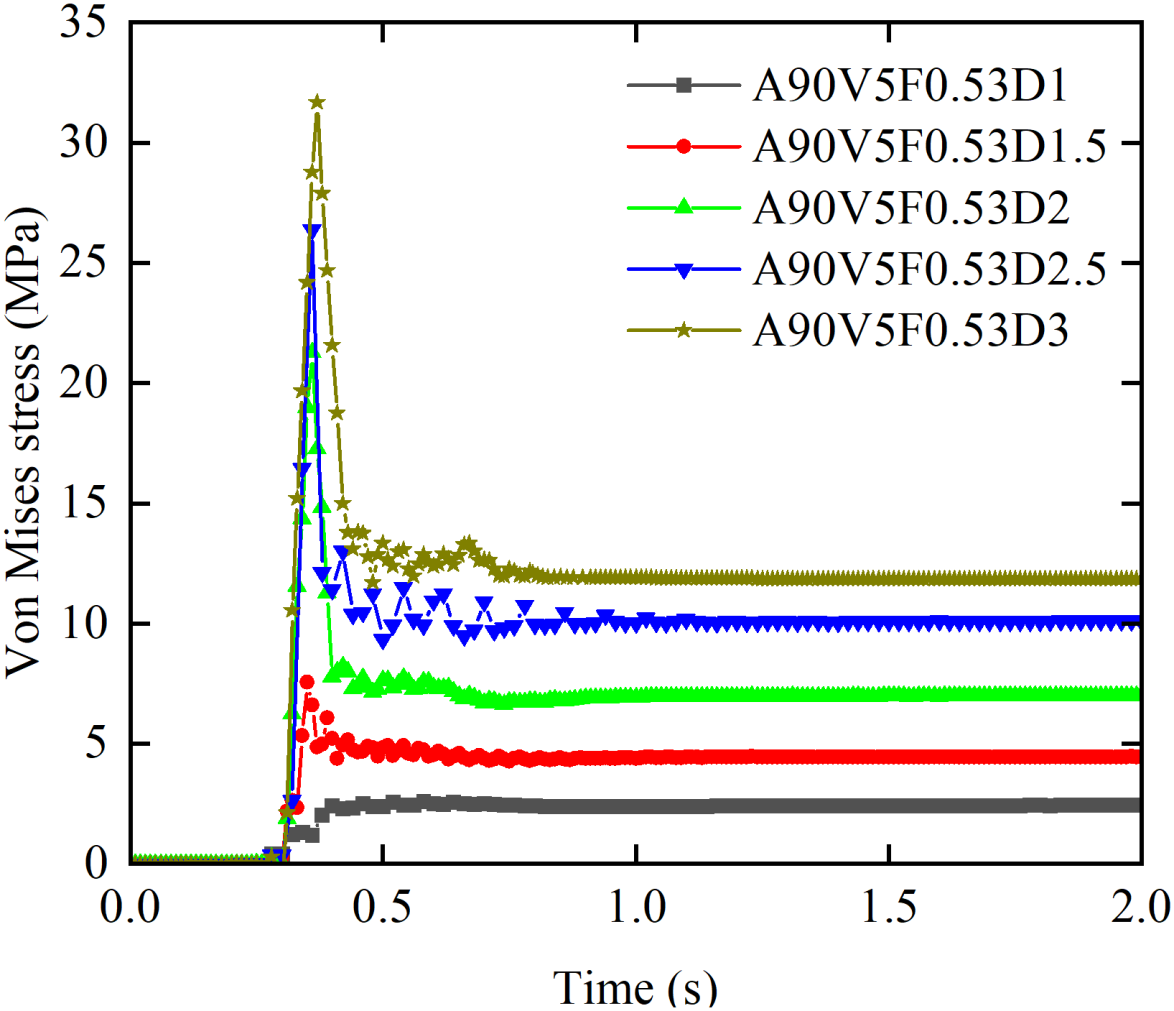
Effect of flow depth on von Mises stress time history curves.

Fig 16 shows the effect of flow depth on von Mises stress time history curves. When boulder impacts are excluded, the stress evolution is similar to the trend shown in Fig 9. The peak von Mises stress (*σ*_peak_) increases 12-fold as the depth increases from 1 to 3 m, at a greater rate than the impact force. This demonstrates that the flow depth enhances the structural response. The steady state stress (*σ*_steady_) increases by a factor of 3.9. The growth rate of *σ*_peak_ with depth is 10.22 MPa/m for depths of 1.5 m or less, 27 MPa/m for depths between 1.5 m and 2 m, and 5.2 MPa/m for depths between 2 m and 3 m. The value of *σ*_peak_ reaches 4.47 times the design compressive strength at a depth of 1.5 m, indicating a risk of localized crushing.

### 3.3 Displacement

#### 3.3.1 Effect of flow velocity on structural displacement.

**Fig 17:**
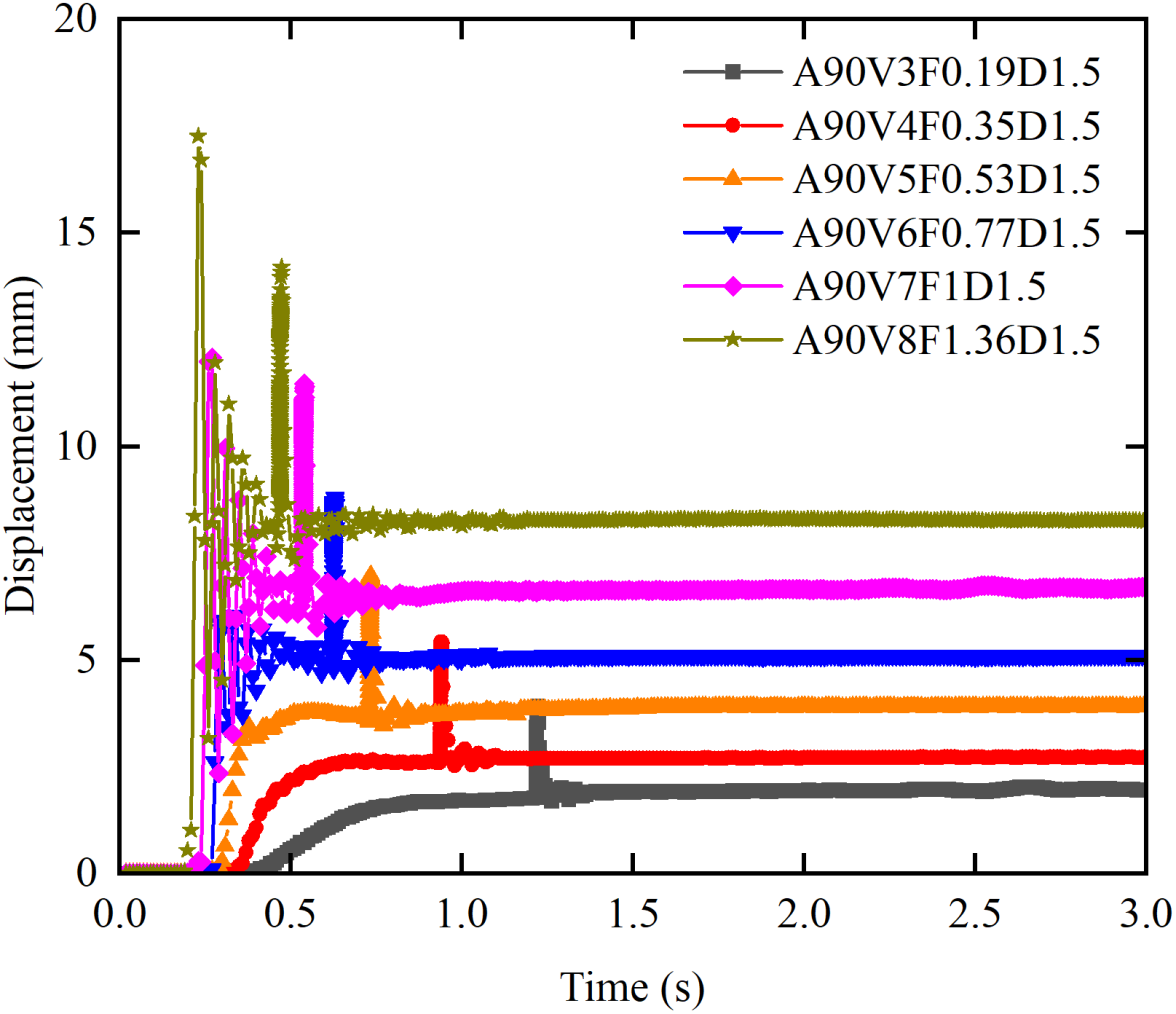
Effect of flow velocity on displacement time history curves for masonry structures.

Fig 17 illustrates the effect of flow velocity on the displacement time history. At flow velocities of 5 m/s or above, the displacement time history of the masonry structure exhibits seven phases and a bimodal response, which reveals a synergistic effect between the slurry and boulder impacts. The slurry impact generates the primary peak (*u*_slurry_), whose time to peak decreases with increasing flow velocity, whereas the boulder impact produces a secondary peak (*u*_boulder_) that exhibits a consistent time lag relative to *u*_slurry_. This lag is synchronized with the delay in the boulder impact force. When the flow velocity exceeds 6 m/s, *u*_slurry_ exceeds *u*_boulder_, and the peak displacement occurred during the slurry phase; in contrast, at velocities of 6 m/s or less, *u*_boulder_ exceeds *u*_slurry_, and the peak displacement occurs during the boulder phase. At flow velocities of 5 m/s or less, *u*_boulder_ is approximately equal to *u*steady. The bimodal response begins at 5 m/s, and a transition in the dominant peak occurs at 6 m/s. As the velocity increases from 3–8 m/s, *u*_slurry_ increases by 13.35 mm, which is a factor of 3.4; *u*_steady_ increases by 6.28 mm, corresponding to a factor of 3.3; and *u*_boulder_ increases by 10.3 mm, representing a factor of 2.6.

#### 3.3.2 Effect of flow depth on structural displacement.

**Fig 18:**
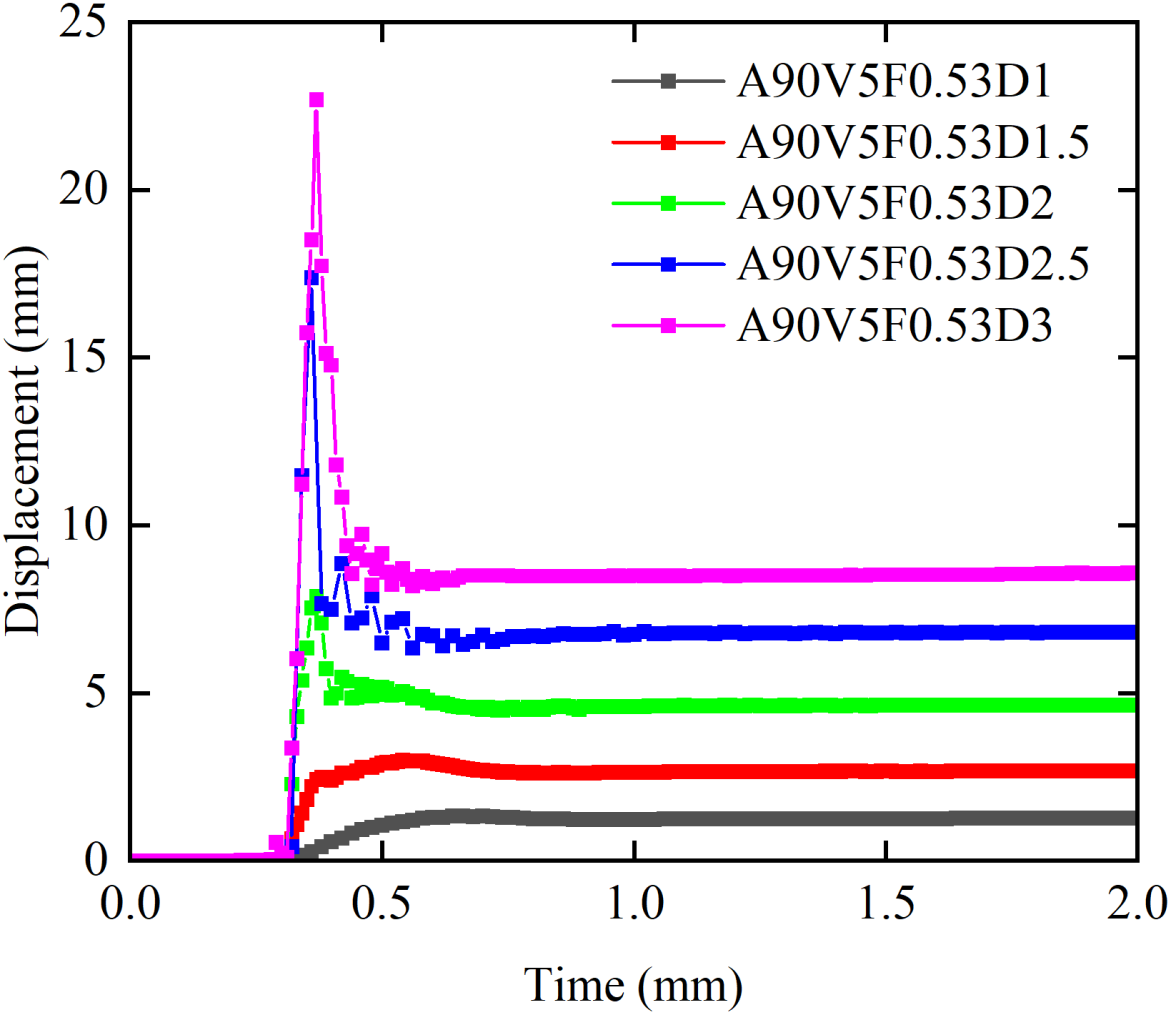
Effect of flow depth on displacement time history curves for masonry structures.

Fig 18 illustrates the effect of flow depth on the displacement time history. Without boulder impacts, the displacement patterns resemble those shown in Fig 9 (impact force) and Fig 14 (von Mises stress), confirming consistent Fluid-Structure Interaction. *u*_peak_ is approximately equal to *u*_steady_ at flow depths of 1.5 m or less. When the flow depth exceeds 1.5 m, the growth of *u*_peak_ accelerates, increasing the difference from *u*_steady_. As the flow depth increases from 1 m to 3 m, *u*_peak_ increases by 21.25 mm, an increase of 14.65 times. The growth rate of the peak displacement is 1.71 times that of the peak impact force, indicating that the displacement response is more susceptible to flow depth variations than the impact force. Concurrently, *u*_steady_ increases by 7.28 mm, an increase of 5.8 times. Both the peak and the steady state displacement exhibit a monotonically increasing trend with flow depth.

## 4. Discussion

### 4.1 Models for displacement prediction


$$u_{peak}=0.448V^{\text{2}}-2.3947V+7.3842$$ (1)

$$u_{steady}=0.1041V^{\text{2}}+0.1248V+0.6289$$ (2)

$$u_{boulder}=0.1864V^{\text{2}}-0.0079V+2.32$$ (3)

Based on the displacement data, three predictive models were formulated: Equation (1) for the overall peak displacement, Equation (2) for the steady state displacement, and Equation (3) for the peak displacement induced by boulders. Displacements were calculated using these models under an impact angle of 90° and a flow depth of 1.5 m. In these models, *u*_peak_, *u*_steady_, *u*_boulder_, and *V* denote the overall peak displacement, the steady state displacement, the peak displacement induced by boulders, and flow velocity, respectively. The predicted displacements for flow velocities ranging from 9 to 15 m/s are summarized in Table 6.

**Table 6:** Displacement of the masonry structure at flow velocities ranging from 9 to 15 m/s.

*V* (m/s)	Peak displacement (mm)	Steady state displacement (mm)	Peak displacement induced by boulder impact (mm)
**9**	22.12	10.18	17.35
**10**	28.24	12.29	20.88
**11**	35.25	14.60	24.79
**12**	43.16	17.12	29.07
**13**	51.97	19.84	33.72
**14**	61.67	22.78	38.74
**15**	72.26	25.92	44.14

### 4.2 Assessment of structural damage

Table 8 summarizes the damage states of masonry structures under various flow conditions. Damage states were characterized using the inter-story displacement angle criteria from Su et al. [38], as detailed in Table 7. Under conditions of a flow velocity not exceeding 3 m/s (with a 90° impact angle and 1.5 m depth), the first story sustained minor damage, the second story was intact, and the boulder impact zone sustained moderate damage. At a flow velocity of 4 m/s, the first story sustained moderate damage and the second story sustained minor damage, while the boulder impact zone sustained severe damage. At flow velocities of 5 m/s or greater, the overall structure sustained moderate damage, while the boulder impact zone sustained partial collapse, as the simulated displacement angle exceeded the collapse limit of 1/150 (Table 8). At a flow velocity of 8 m/s, the first story sustained severe damage, and the second story sustained moderate damage. Thus, a flow velocity of 5 m/s was identified as the critical velocity for partial collapse, and 8 m/s was the critical velocity for severe damage in the first story. These critical thresholds provide actionable criteria for disaster early warning and the design of protective measures in debris-flow-prone regions.

**Table 7:** Limitations of inter-story displacement angle for different damage states of masonry structures [38].

Structural damage state	Complete	Slight damage	Severe damage	Collapse
**Inter-story displacement angle**	1/2500	1/2000	1/200	1/150

**Table 8:** Damage states of masonry structures.

Conditions	First story			Second story			Boulder impact zone		
	Inter-story displacement (mm)	Inter-story displacement angle	Damage states	Inter-story displacement (mm)	Inter-story displacement angle	Damage states	Displacement (mm)	Inter displacement angle	Damage states
**A90V3F0.19D1.5**	1.47	1/2108	Slight damage	1.93	1/3212	Complete	3.9	1/256	Moderate damage
**A90V4F0.35D1.5**	1.9	1/1631	Moderate damage	2.69	1/2304	Slight damage	5.42	1/184	Severe damage
**A90V5F0.53D1.5**	2.58	1/1201	Moderate damage	3.9	1/1589	Moderate damage	6.98	1/143	Collapse
**A90V6F0.77D1.5**	3.73	1/831	Moderate damage	5.1	1/1212	Moderate damage	8.85	1/112	Collapse
**A90V7F1D1.5**	9.63	1/321	Moderate damage	12.1	1/512	Moderate damage	11.7	1/85	Collapse
**A90V8F1.36D1.5**	15.8	1/196	Severe damage	17.3	1/385	Moderate damage	14.1	1/71	Collapse
**A90V5F0.53D1**	1.12	1/2767	Complete	1.24	1/5000	Complete	——		
**A90V5F0.53D1.5**	3.51	1/883	Moderate damage	4.47	1/1387	Moderate damage			
**A90V5F0.53D2**	9.38	1/330	Moderate damage	12.55	1/494	Moderate damage			
**A90V5F0.53D2.5**	13.56	1/228	Moderate damage	17.38	1/356	Moderate damage			
**A90V5F0.53D3**	17.81	1/174	Severe damage	22.29	1/278	Moderate damage			

Damage severity increased with flow depth. Under a 90° impact angle and a flow velocity of 5 m/s without boulders, structural damage increased with depth. No damage occurred at 1 m depth; moderate damage was sustained at 1.5 m depth; and at 3 m depth, the first story sustained severe damage, while the second story sustained moderate damage. Consequently, a depth of 3 m was identified as the critical threshold for severe structural damage.

Two displacement prediction models were developed: Equation (4) for the first story displacement, *u*_floor1_, and Equation (5) for second story displacement, *u*_floor2_. These models are applicable at an impact angle of 90° and a flow depth of 1.5 m. As shown in Table 9, the displacement angle exceeded the collapse limits at flow velocities of 9 m/s and above, thereby establishing a flow velocity of 9 m/s as the critical velocity for structural collapse.

**Table 9:** Damage states of masonry structures at flow velocities ranging from 9 to 15 m/s.

*ν* (m/s)	First story			Second story		
	Inter-story displacement (mm)	Inter-story displacement angle	Damage states	Inter-story displacement (mm)	Inter-story displacement angle	Damage states
**9**	23.72	1/135	Collapse	25.36	1/126	Collapse
**10**	33.54	1/95		34.88	1/92	
**11**	45.14	1/71		46.01	1/70	
**12**	58.51	1/55		58.77	1/54	
**13**	73.65	1/43		73.14	1/44	
**14**	90.56	1/35		89.14	1/36	
**15**	109.24	1/29		106.76	1/30	


$$u_{floor1}=0.8854V^{\text{2}}-6.9964V+14.967$$ (4)

$$u_{floor2}=0.81V^{\text{2}}-5.8734V+2.609$$ (5)

### 4.3 Influence of boulders on dynamic response and failure mechanisms

**Fig 19:**
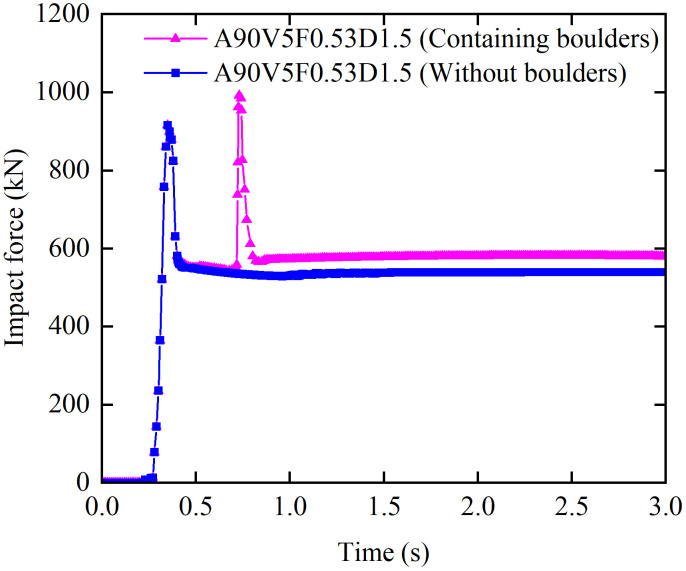
Impact force time history for debris flows with and without boulders under Condition A90V5F0.53D1.5.

Fig 19 compares the impact force time history for debris flows with and without boulders under Condition A90V5F0.53D1.5 (impact angle: 90°, flow velocity: 5 m/s, Froude number: 0.53, flow depth: 1.5 m). For the debris flow without boulders, the impact force time history curve exhibits a single peak (*F*slurry), which is generated by the slurry. In contrast, a debris flow containing boulders exhibits a distinct bimodal profile (Fig 19). The initial peak (*F*slurry) corresponds to the impact of the slurry front, while the secondary peak (*F*boulder) corresponds to the concentrated impact of entrained boulders (Fig 19). This bimodal response indicates that the structure experiences distributed loading from the slurry followed by concentrated impact loading from boulders. This sequence results in a complex stress state characterized by sequential surface contact and point impacts, as well as the superposition of dynamic and static forces.

In contrast to the decaying pattern following a single impact peak in debris flows without boulders, boulder induced secondary peaks typically exhibit higher impact forces that persist for a shorter duration. The resulting instantaneous stress concentration readily causes shear failure near the impact area in masonry structures. Furthermore, the high energy introduced by boulder impacts significantly increases von Mises stress within the structure and exacerbates global deformation. This combination accelerates the progression from localized damage to global instability, consequently reducing the impact resistance of masonry walls and influencing their final collapse modes. These findings unequivocally demonstrate that boulders are a dominant factor in altering debris flow-structure interaction patterns and triggering catastrophic structural failure mechanisms.

Quantitative comparisons of the dynamic response parameters are summarized in Table 10, Which demonstrates the significant influence of boulders on the dynamic response of masonry structures, showing no significant differences in the peak impact force (*F*_slurry_), peak von Mises stress (*σ*_slurry_), and peak displacement (*u*_slurry_) induced by the slurry under the two conditions, whereas the presence of boulders significantly amplifies all subsequent response metrics. The peak impact force from boulders (*F*_boulder_) exceeds that from the slurry (*F*_slurry_) by 8.22%, indicating that boulders act as concentrated loads that intensify instantaneous impact effects. More notably the peak von Mises stress induced by boulders (*σ*_boulder_) is 114.29% higher than that induced by the slurry (*σ*_slurry_). This increase significantly exceeds the relative growth in impact force; and stress analysis reveals pronounced stress concentrations within the masonry structure that may trigger local shear failure. Additionally, boulders also enhance the steady state response: the steady state impact force (*F*_steady_) increases by 7.38%, and the steady state von Mises stress (*σ*_steady_) rises by 1.44 N/mm² compared to the case without boulders. This indicates that boulders exacerbate sustained loading and generate considerable residual stress, thereby compromising long term structural safety. Regarding displacement, the peak displacement induced by boulders (*u*_boulder_) is 87.40% greater than that induced by the slurry (*u*_slurry_). Moreover, the steady state displacement (*u*_steady_) containing boulders is 1.23 mm greater than that without boulders. These results confirm that boulders not only aggravate instantaneous deformation but also induce greater irreversible displacement, thereby accelerating the progression from localized damage to global structural instability. For a more in-depth and specialized analysis on the impact of high-velocity boulders on structures can be found in Reference 15.

**Table 10:** Comparison of dynamic responses for debris flows with and without boulders under Condition A90V5F0.53D1.5.

Debris flow	Impact force (kN)			Von Mises stress (MPa)			Displacement (mm)		
	*F*_slurry_	*F*_boulder_	*F*_steady_	*σ*_slurry_	*σ*_boulder_	*σ*_steady_	*u*_slurry_	*u*_boulder_	*u*_steady_
**Containing boulders**	915.69	991	579	7.56	16.2	5.89	3.73	6.99	3.9
**Without boulders**	915.69	—	539.2	7.56	—	4.45	3.73	—	2.67

*F*_slurry_, *F*_boulder_, and *F*_steady_ represent the peak impact force from slurry, the peak impact force from boulders, and the steady state impact force, respectively. *σ*_slurry_, *σ*_boulder_, and *σ*_steady_ represent the peak von Mises stress from slurry, the peak von Mises stress from boulders, and the steady state von Mises stress, respectively. *u*_slurry_, *u*_boulder_, and *u*_steady_ represent the peak displacement from slurry, the peak displacement from boulders, and the steady state displacement.

### 4.4 Comparative analysis of numerical simulation and field investigation

To evaluate the reliability and predictive accuracy of our Fluid-Structure Interaction (FSI) model, we conducted a comparative analysis between the numerical simulations and field observations of masonry structure damage. The comparison focuses on three aspects: failure modes, critical failure states, and mechanical response mechanisms.

#### 4.4.1 Comparison of simulated and observed failure modes.

Field investigations revealed three primary failure modes in the affected masonry structures (Fig 3). Foundation erosion failure was caused by scouring from debris flow containing boulders, leading to differential settlement. Impact shear failure involved the localized collapse of first-story load-bearing walls from the direct impact of boulders. Global overturning and collapse occurred when the lateral fluid thrust exceeded the structure’s overturning moment resistance.

Under the working condition of A90V5F0.53D1.5, the masonry structure exhibited a local collapse failure mode in the boulder impact zone. The maximum von Mises stress reached 16.45 MPa, which is 9.73 times the masonry’s design compressive strength (1.69 MPa). This finding confirms the impact-induced shear failure mode documented in field investigations. Furthermore, at an increased flow velocity of 9 m/s, the inter-story displacement angle surpassed the collapse limit of 1/150, indicating global structural instability (Table 8). This numerical result is consistent with the global overturning collapse observed in the field.

#### 4.4.2 Comparative analysis of critical failure states.

Using the inter-story displacement angle limits of masonry structures (Table 7), Table 8 summarizes the failure states under various working conditions. Field investigations indicate that the displacement characteristics corresponding to slight, moderate damage, and collapse in masonry structures match simulation results. At a flow velocity of 5 m/s, the simulated displacement at the boulder impact point reached 6.98 mm, yielding a displacement angle of 1/143 that exceeded the collapse limit. This simulated failure state matches the partial collapse observed in the actual disaster (Fig 3). At a flow velocity of 8 m/s, the simulated inter-story displacement angle of the first story masonry structure reached 1/196, classified as severe damage. The numerical simulation results are closely aligned with the field investigations in Fig 3, particularly regarding the multiple cracks and localized collapse patterns observed on the structural surface. The consistency between numerical and field investigations demonstrates the practical engineering applicability of the inter-story displacement angle. It also confirms that the model can effectively predict damage extent in masonry structures under varying flow velocities.

#### 4.4.3 Comparison analysis of mechanical response mechanisms.

The numerical results in Figs 7 and 8 show a dual-peak characteristic in the impact force time history. This pattern reflects the mechanistic sequence of failure observed in the field: the structure is first impacted by debris flow slurry, followed by boulders. The peak slurry impact force (*F*_slurry_) induces the initial stress peak, which causes surface cracking and degrades wall stiffness. The subsequent boulder impact force (*F*_boulder_) then acts on this pre-damaged structure. This simulated failure progression aligns with field observations of progressive spalling from the exterior to the interior, a process that culminates in wall collapse. Moreover, the simulated stress concentration at the structure’s base (Figs 11 and 12) corresponds directly to the foundation scouring and collapse mechanisms documented in the field. This further validates the effectiveness of the numerical model in revealing force transmission pathways and the mechanism of local damage evolution.

In summary, the numerical results agree with field investigations across three key aspects: failure modes, critical failure states, and mechanical response mechanisms of masonry structures. These findings validate the accuracy of the Fluid-Structure Interaction model and its capability to predict dynamic responses and failure patterns of masonry structures under debris flow impacts containing boulders. It provides a scientific basis for disaster assessment and protective design of masonry structures in rural areas.

### 4.5 Limitations and future work

The current study has several limitations. Specifically, the effects of boulder geometry are not quantified, cumulative damage from sequential impacts is not analyzed, and the range of investigated impact angles is limited. To address these issues, subsequent research will employ a coupled SPH-DEM-FEM multiscale model. This approach will elucidate the mechanisms of stochastic energy transfer during impact and, crucially, will enable the quantification of both boulder geometry effects and cumulative damage. Furthermore, based on boulder response spectrum theory, vulnerability surfaces for rural masonry structures will be developed. These surfaces will incorporate the randomness of boulder impacts and the accumulation of structural damage.

## 5. Conclusions

This study has developed a Fluid-Structure Interaction (FSI) model to simulate the dynamic responses and failure mechanisms of rural masonry structures impacted by debris flow containing boulders. The main conclusions are as follows:

(1) The impact force from debris flows containing boulders exhibits a distinct bimodal pattern with a time lag. At an impact angle of 90° and a flow depth of 1.5 m, the impact force undergoes seven phases with bimodal characteristics emerging when the flow velocity reaches or exceeds 4 m/s. The primary peak (*F*_slurry_) originates from the slurry impact, while the secondary peak (*F*_boulder_) arises from boulder impact. The time lag of the secondary peak is inversely proportional to the flow velocity. The dominance of the force peaks transitions at a flow velocity of 5 m/s: slurry induced peaks dominate above this threshold, whereas boulder induced peaks prevail at lower velocities. Impact forces scale nonlinearly with flow depth, inducing foundation erosion.
(2) The dynamic response depends on the flow velocity. Both von Mises stress and displacement time history curves exhibit a dual peak pattern at flow velocities of 5 m/s or higher, and a single peak pattern at lower velocities. The peak values, steady state values, and the overall profile of the von Mises stress time history curves, particularly those induced by boulder impact, are significantly influenced by increasing flow velocity and depth. Boulder impact leads to significant stress concentration within localized regions of the masonry structure, while concurrently triggering a nonlinear surge in the global structural stress levels. This nonlinear stress surge significantly exacerbates cumulative structural damage under debris flow impact, thereby increasing the risk of global instability or even catastrophic collapse.
(3) Critical flow velocities for structural collapse are identified. Under the specified conditions of a 90° impact angle and 1.5 m flow depth, a flow velocity of 5 m/s causes partial collapse within the boulder impact zone, establishing 5 m/s as the critical flow velocity for partial structural collapse. Based on these findings, two critical flow velocities are identified: 5 m/s for the initiation of partial collapse and 9 m/s for global structural collapse.
(4) A critical flow depth for severe damage is established. At an impact angle of 90° and a flow velocity of 5 m/s, severe damage is observed in the first story of the masonry structures when the flow depth reaches 3 m. Therefore, under these conditions, the critical flow depth for severe structural damage is identified as 3 m.
(5) Based on inter-story displacement angle analysis, predictive formulas for displacement and a corresponding damage classification standard are developed for masonry structures. These results provide a practical basis for assessing disaster risk and planning reinforcement strategies for vulnerable structures.
(6) The model is verified against field data, demonstrating its reliability and practical utility. Numerical results show strong consistency with field surveys in terms of failure modes, critical states, and mechanical mechanisms, validating the model’s accuracy and engineering suitability. These findings provide a scientific basis for protective design in debris-flow hazard zones.
